# Co‐staining of K_Ca_3.1 Channels in NSCLC Cells with a Small‐Molecule Fluorescent Probe and Antibody‐Based Indirect Immunofluorescence

**DOI:** 10.1002/cmdc.202000652

**Published:** 2020-10-21

**Authors:** Kathrin Brömmel, Sarah Maskri, Etmar Bulk, Zoltan Pethő, Marius Rieke, Thomas Budde, Oliver Koch, Albrecht Schwab, Bernhard Wünsch

**Affiliations:** ^1^ Institute for Pharmaceutical and Medicinal Chemistry Westphalian Wilhelms-University Münster Corrensstraße 48 48149 Münster Germany; ^2^ Institute for Physiology II University Hospital Münster Robert-Koch-Straße 27b 48149 Münster Germany; ^3^ Institute for Physiology I University Hospital Münster Robert-Koch-Straße 27a 48149 Münster Germany; ^4^ Cells-in-Motion Interfaculty Center Westphalian Wilhelms-University Münster Waldeyerstraße 15 84149 Münster Germany

**Keywords:** KCa3.1 channel, non-small cell lung cancer cells, senicapoc derivatives, fluorescent probes, co-staining

## Abstract

The Ca^2+^ activated potassium channel 3.1 (K_Ca_3.1) is involved in critical steps of the metastatic cascade, such as proliferation, migration, invasion and extravasation. Therefore, a fast and efficient protocol for imaging of K_Ca_3.1 channels was envisaged. The novel fluorescently labeled small molecule imaging probes **1** and **2** were synthesized by connecting a dimethylpyrrole‐based BODIPY dye with a derivative of the K_Ca_3.1 channel inhibitor senicapoc via linkers of different length. Patch‐clamp experiments revealed the inhibition of K_Ca_3.1 channels by the probes confirming interaction with the channel. Both probes **1** and **2** were able to stain K_Ca_3.1 channels in non‐small‐cell lung cancer (NSCLC) cells following a simple, fast and efficient protocol. Pre‐incubation with unlabeled senicapoc removed the punctate staining pattern showing the specificity of the new probes **1** and **2**. Staining of the channel with the fluorescently labeled senicapoc derivatives **1** or **2** or with antibody‐based indirect immunofluorescence yielded identical or very similar densities of stained K_Ca_3.1 channels. However, co‐staining using both methods did not lead to the expected overlapping punctate staining pattern. This observation was explained by docking studies showing that the antibody used for indirect immunofluorescence and the probes **1** and **2** label different channel populations. Whereas the antibody binds at the closed channel conformation, the probes **1** and **2** bind within the open channel.

## Graphical abstract

**Open vs. closed**: The K_Ca_3.1 channel is involved in critical steps of the metastatic cascade. Analyzing K_Ca_3.1 channel expression has predictive power with respect to patient survival. Novel fluorescently labeled small‐molecule imaging probes were able to stain K_Ca_3.1 channels in NSCLC cells selectively and efficiently. Co‐staining using antibody‐based indirect immunofluorescence did not lead to the expected overlapping punctate staining pattern. This observation was explained by docking studies showing that the antibody and the novel imaging probes label different channel populations, closed and open channels, respectively.

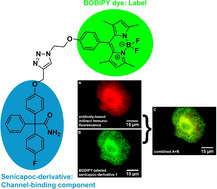

## Introduction

Ion channels contribute to features of essentially all “cancer hallmarks”.^[1]^ The Ca^2+^ activated potassium channel 3.1 (K_Ca_3.1) is involved in critical steps of the metastatic cascade, such as proliferation, migration, invasion and extravasation.[^2^, ^3^] Inhibition of the K_Ca_3.1 channel by different small molecules leads in many different tumor entities to reduced proliferation, migration and metastasis.[^4^, ^5^, ^6^] Moreover, overexpression of K_Ca_3.1 channels and tumor grade as well as metastatic status correlate with each other so that elevated K_Ca_3.1 expression is often related to poor prognosis of tumor patients.^[7]^ Thus, analyzing K_Ca_3.1 channel expression has predictive power with respect to patient survival.[^8^, ^9^] Additionally it has been reported that endogenously expressed K_Ca_3.1 channels have pro‐tumor functions in a mouse model.[^10^, ^11^]

**Figure 1:**
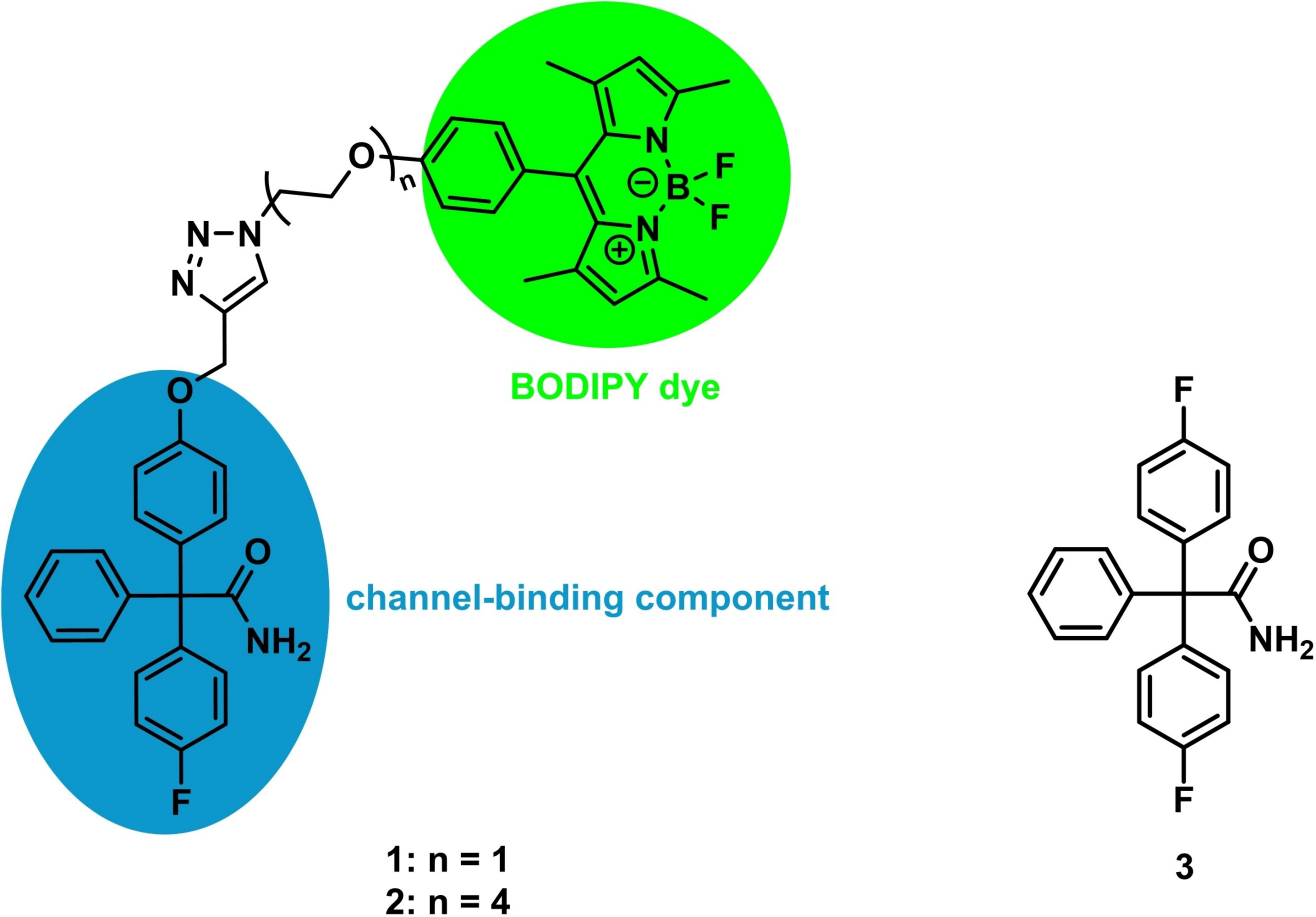
BODIPY‐labeled senicapoc derivatives **1**, **2** and senicapoc (**3**).

Recently we published the development and synthesis of novel fluorescently labeled small‐molecule probes as tools for fast and efficient visualization of K_Ca_3.1 channels.^[12]^ Best results were achieved with compounds **1** and **2** (corresponding to the fluorescently labeled ligands previously reported),^[12]^ which are dimethylpyrrole‐based BODIPY‐labeled derivatives of the K_Ca_3.1 channel inhibitor senicapoc (**3**). (Figure 1).

**Figure 2:**
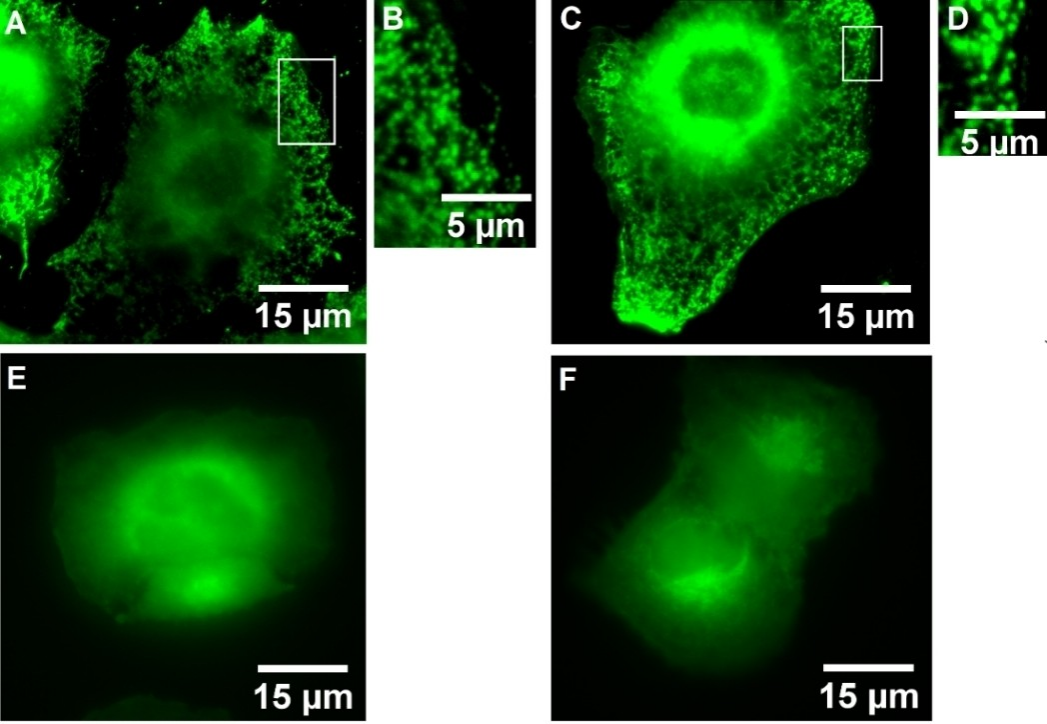
**A**: A549‐3R cells incubated for 10 min with a 10 μM staining solution of BODIPY‐labeled senicapoc derivative **1. B**: Magnification of **A** (white box). **C**: A549‐3R cells incubated for 10 min with a 10 μM staining solution of BODIPY‐labeled senicapoc derivative **2. D**: Magnification of **C** (white box). **E**: NSCLC cells blocked for 5 min with unlabeled Senicapoc (30 μM) and subsequently stained for 10 min with a 10 μM staining solution of **1. F**: NSCLC cells blocked for 5 min with unlabeled Senicapoc (30 μM) and subsequently for 10 min with a 10 μM staining solution of **2**.

Senicapoc (**3**) is a highly potent and selective inhibitor of the K_Ca_3.1 channel (IC_50_=11±2 nM; measured on human erythrocytes).^[13]^ The senicapoc part of **1** and **2** is the targeting component of the small molecules. The BODIPY‐labeled senicapoc derivatives **1** and **2** showed the punctate staining pattern of the K_Ca_3.1 channel in NSCLC cells after only 10 min incubation time. (Figure 2) Pre‐incubation with senicapoc blocked all binding sites for **1**, so that the punctate staining pattern was not observed any more. The densities of K_Ca_3.1 channel‐related dots resulting from staining with **1** and from an antibody‐based indirect immunofluorescence assay were identical.^[12]^ However, co‐staining was not successful when both staining protocols were performed sequentially. Therefore, a new protocol was developed that allowed co‐staining using both protocols.

Moreover, we performed patch‐clamp experiments with **1** using A549‐3R cells to investigate the inhibitory effect of **1** towards the K_Ca_3.1 channel.

## Results

The typical punctate staining pattern of the K_Ca_3.1 channel was observed after incubation of the A549‐3R cells with a 10 μM staining solution of **1** for 10 min.[^7^, ^12^, ^14^] (Figure 2A,B) The same staining pattern was obtained after incubation of the cells with the BODIPY‐labeled senicapoc derivative **2** which has a longer linker (four OCH_2_CH_2_ units) between the targeting component and the BODIPY dye. (Figure 2C,D) The most likely explanation for the strong perinuclear staining is the labeling of intracellularly located channels (e. g. channels in the ER, Golgi and/or mitochondria). A similar pattern was also observed when cells were transfected with a GFP‐labeled KCa3.1 channel.^[15]^ As described, pre‐incubation with senicapoc eliminated the punctate staining pattern of **1** and **2** confirming that the BODIPY‐labeled senicapoc derivatives **1** and **2** label selectively K_Ca_3.1 channels.^[12]^ (Figure 2 E–F)

**Figure 3:**
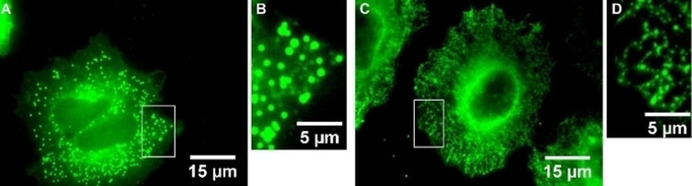
**A**: A549‐3R cells permeabilized with Triton X‐100 solution and subsequent incubation with a 10 μM staining solution of BODIPY‐labeled senicapoc derivative **1** for 10 min. **B**: Magnification of **A** (white box). **C**: A549‐3R cells permeabilized with saponine solution and subsequent incubation with a 10 μM staining solution of BODIPY‐labeled senicapoc derivative **1** for 10 min. **D**: Magnification of **C** (white box).

When A549‐3R cells were permeabilized with Triton X‐100 solution first and then incubated with a 10 μM staining solution of **1**, the resulting staining pattern changed considerably. The punctate signals showed a ***F**ull **W**idth at **H**alf **M**aximum* (FWHM)>5 pixels and can therefore not be interpreted as K_Ca_3.1 channels. Changing the permeabilization to a saponine solution led again to the typical punctate staining pattern of the K_Ca_3.1 channel with FWHM≤5 pixels after incubation of A549‐3R cells with a 10 μM staining solution of **1** for 10 min. (Figure 3)

**Figure 4:**
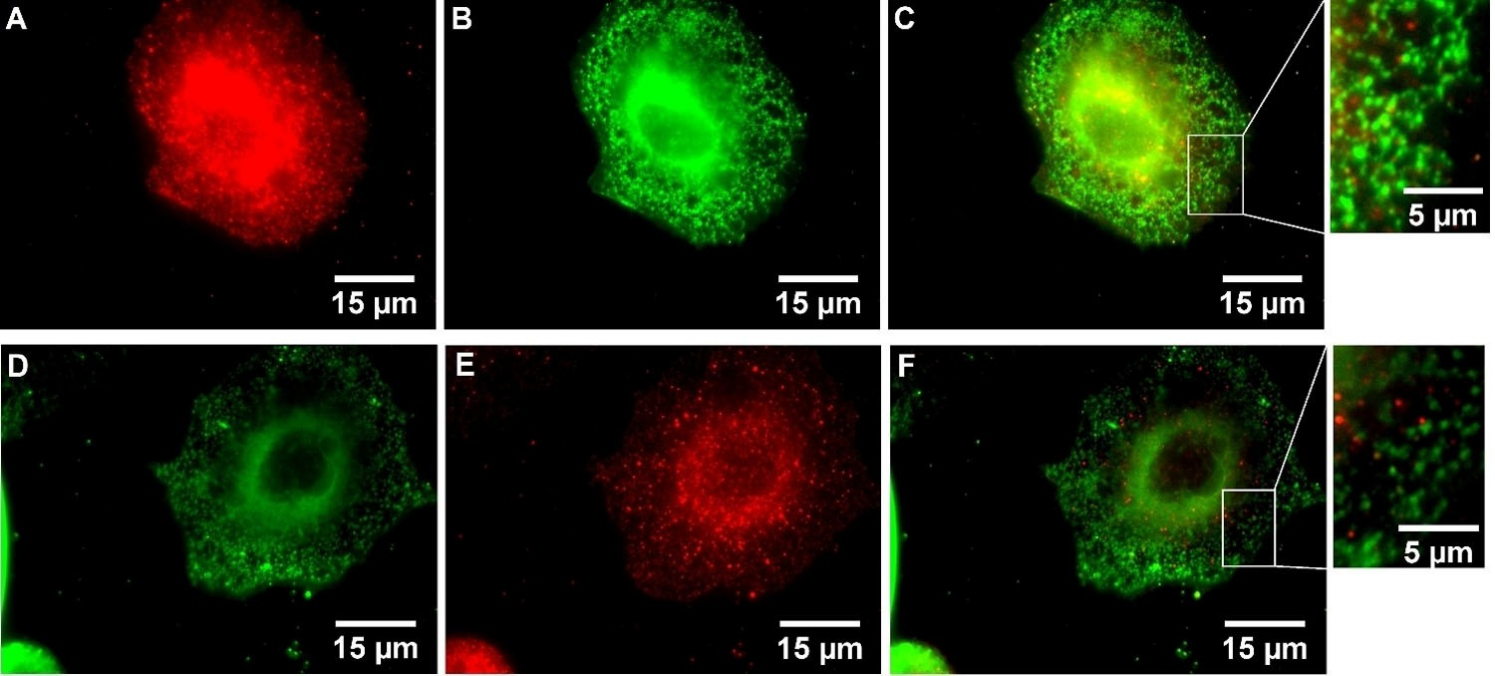
**A**–**C**: A549‐3R cells after performing staining protocol 4B. **A**: antibody‐based indirect immunofluorescence (TRITC filter set). **B**: BODIPY‐labeled senicapoc derivative **1** (FITC filter set). **C**: Combined **A**+**B. D**–**F**: A549‐3R cells after performing staining protocol 5. **D**: BODIPY‐labeled senicapoc derivative **1** (FITC filter set). **E**: antibody‐based indirect immunofluorescence (TRITC filter set). **F**: Combined **D**+**E**.

**Figure 5:**
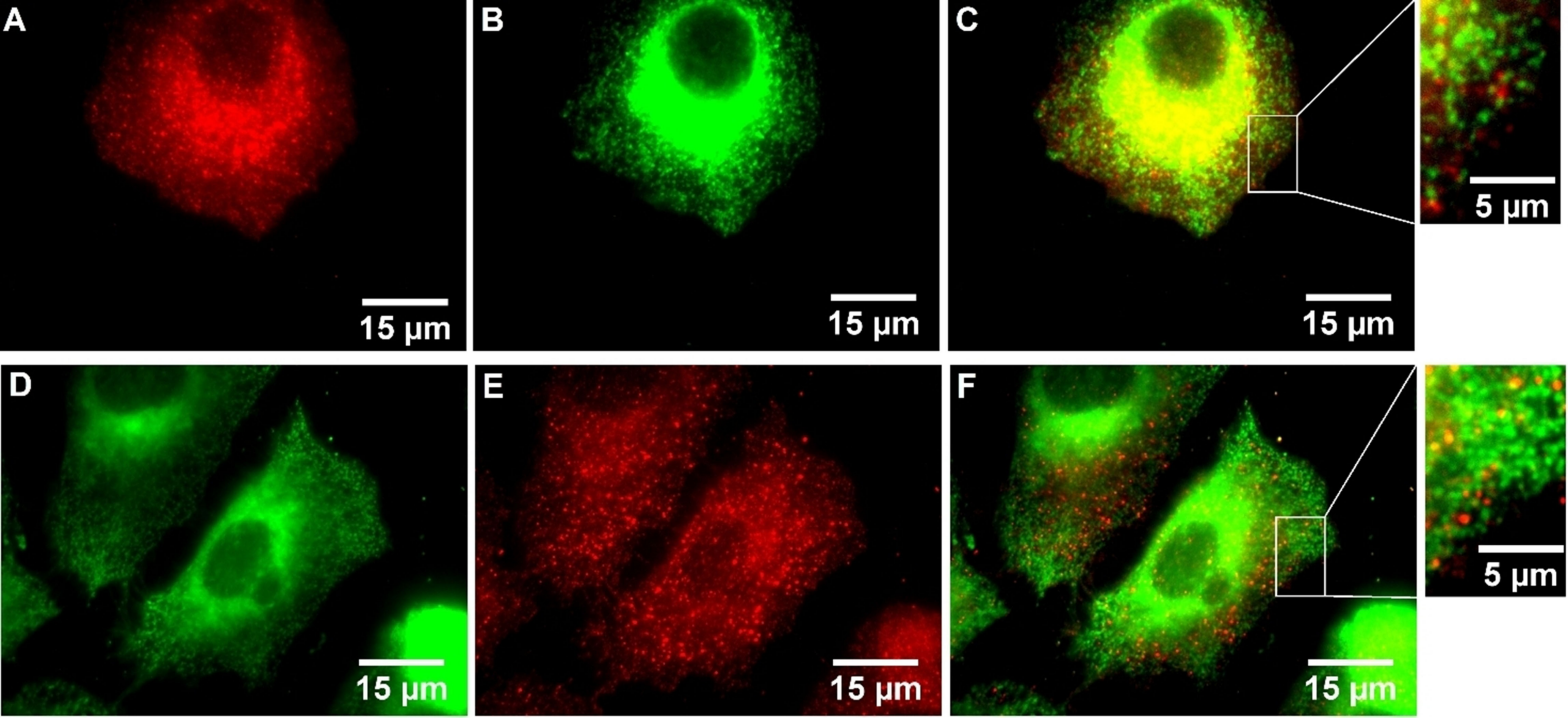
**A**–**C**: **A**–**C**: A549‐3R cells after performing staining protocol 4B. **A**: antibody‐based indirect immunofluorescence (TRITC filter set). **B**: BODIPY‐labeled senicapoc derivative **2** (FITC filter set). **C**: Combined **A**+**B. D**–**F**: A549‐3R cells after performing staining protocol 5. **D**: BODIPY‐labeled senicapoc derivative **2** (FITC filter set). **E**: antibody‐based indirect immunofluorescence (TRITC filter set). **F**: combined **D**+**E**.

After performing the antibody‐based staining and subsequently incubating the cells with the BODIPY‐labeled senicapoc derivatives **1** or **2** for 10 min (protocol 4 A), only the staining of the antibody‐based indirect immunofluorescence was observed with the tetramethylrhodamine isothiocyanate (TRITC) filter set. Signals were not found after switching to the fluorescein isothiocyanate (FITC) filter set (BODIPY‐labeled senicapoc derivatives **1** and **2**). However, extending the incubation time for the BODIPY‐labeled ligand **1** to 20 min (protocol 4B, Figure 4 A–C) led to signals for both filter sets. This method also worked for BODIPY‐labeled senicapoc derivative **2**. (Figure 5 A–C) Likewise, signals for both filter sets were obtained using staining protocol 5 (permeabilization–incubation with **1** or **2**–indirect immunofluorescence, Figures 4 D–F and Figure 5 D–F). Images for both filter sets were combined and evaluated for colocalization of the K_Ca_3.1 channel‐related punctate using the MetaVue software.

**Figure 6:**
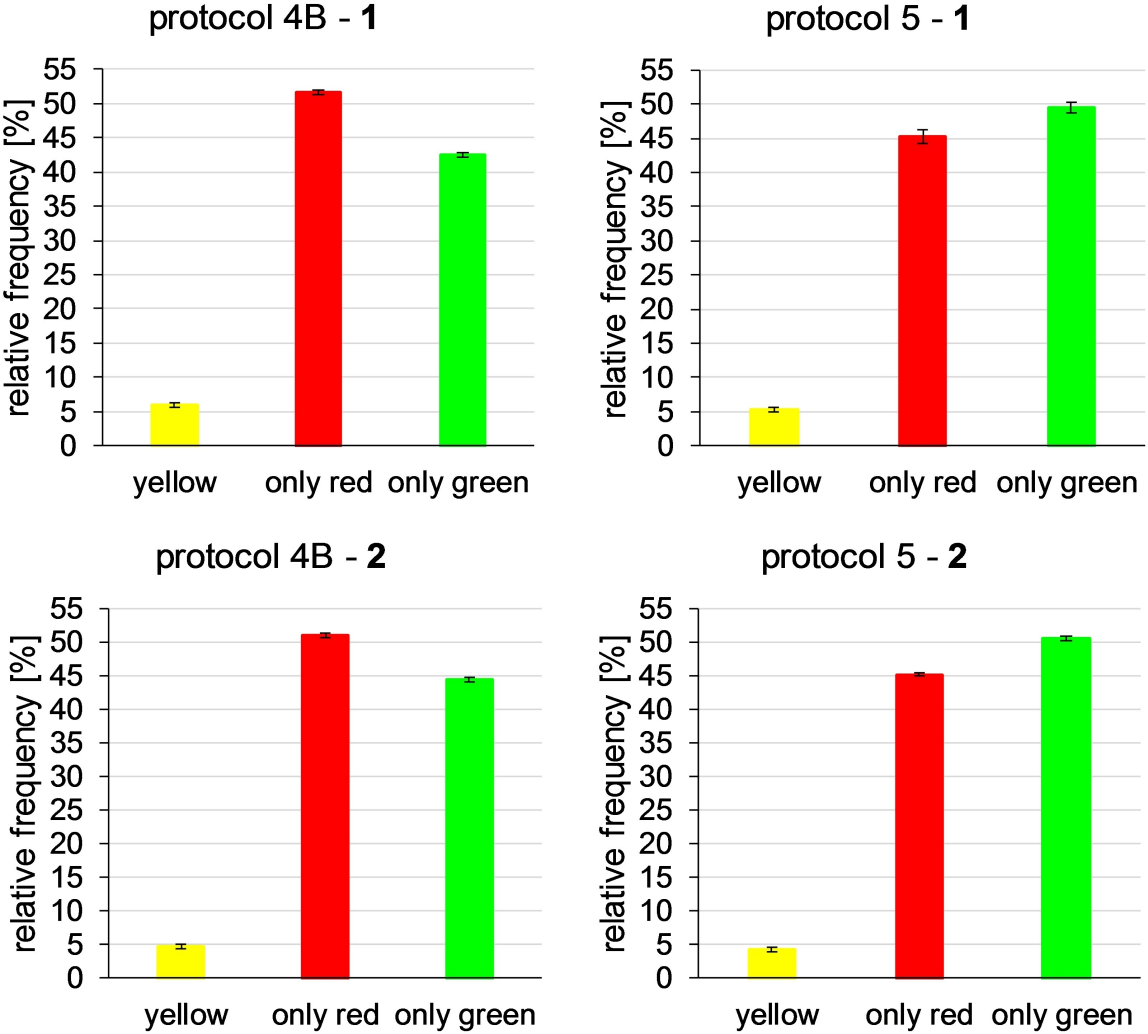
Relative frequency of channel labelling observed in the co‐staining experiments. Protocol 4B: Antibody‐based indirect immunofluorescence staining was performed first followed by subsequent staining with **1** (top) or **2** (bottom). Protocol 5: The permeabilized cells were stained first with the **1** or **2** followed by the antibody‐based indirect immunofluorescence staining.

The density of the K_Ca_3.1 channel‐related punctate signals (only red=antibody‐based immunofluorescence; only green=BODIPY‐labeled senicapoc derivatives; yellow=colocalization) was analyzed by counting the signals in ten squares with a side length of 50 pixels, corresponding to an area of 9 μm^2^, which were randomly placed in a given cell. When a signal was seen in both fluorescence channels, colocalization of the signals required that the x and y coordinates of the “red” and “green” maxima did not differ by more than one pixel.^[14]^ (see supporting information) The results are summarized in Figure 6, Table 1 and Table S2 (Supporting Information).

**Table 1:** K_Ca_3.1 channel density [number of K_Ca_3.1 channels/μm^2^]±SEM.

protocol – compound	channel density			
	only red	only green	yellow	total
IF^[a]^	–	1.79±0.02^[a]^	–	1.79±0.02^[a]^
1‐1	–	1.79±0.02	–	1.79±0.02
1‐2	–	1.69±0.03	–	1.69±0.03
4B‐1	0.95±0.04	0.80±0.03	0.11±0.01	1.86±0.07
5‐1	1.01±0.04	1.10±0.03	0.12±0.01	2.23±0.07
4B‐2	1.07±0.02	0.93±0.02	0.10±0.01	2.09±0.03
5‐2	1.09±0.03	1.22±0.02	0.11±0.01	2.42±0.05

[a] Antibody‐based indirect immunofluorescence.^[12]^

**Figure 7:**
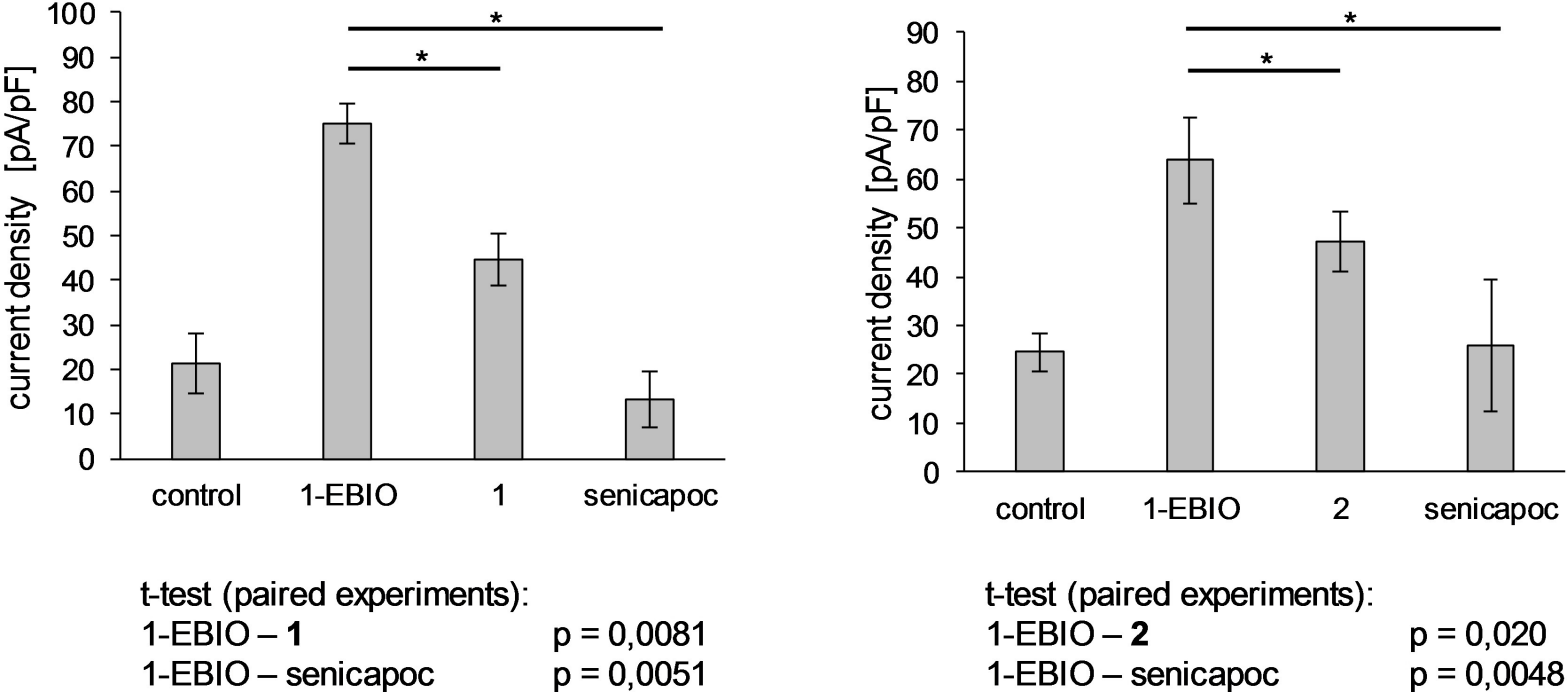
Patch‐clamp experiments. Average current density [pA/pF]± SEM (n=4). 1‐EBIO (50 μM), 1 (10 μM), 2 (10 μM) and senicapoc (1 μM) were dissolved in DMSO and added to the standard extracellular solution and applied in this order for a period of 2–5 min. 1‐EBIO=1‐ethyl‐2‐benzimidazolone.

Additionally, patch‐clamp experiments were performed to investigate the inhibitory activity of the BODIPY‐labeled senicapoc derivatives **1** and **2** towards the K_Ca_3.1 channel (Figure 7).

The experiments were performed in a paired fashion and revealed that both compounds **1** and **2** inhibited K_Ca_3.1 channels, although not as efficiently as senicapoc.

## Discussion

**Figure 8:**
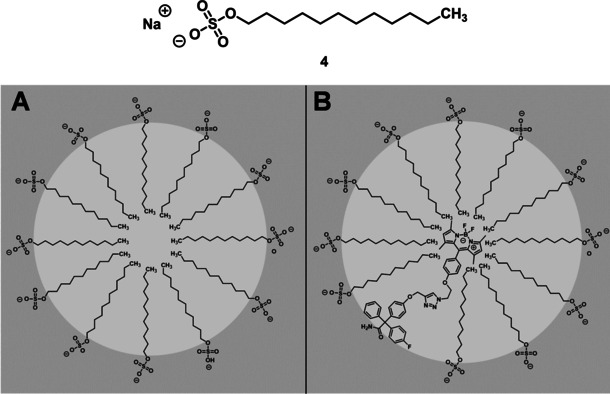
SDS (**4**). **A**: Structure of compound‐free micelles. **B**: Structure of micelles with the embedded BODIPY‐labelled senicapoc derivative **1**.

Since the antibody used here interacts with the intracellular part of the K_Ca_3.1 channel, A549‐3R cells needed to be permeabilized to perform successfully the indirect immunofluorescence staining. In order to perform co‐staining the compatibility of the permeabilization agents and the BODIPY‐labeled senicapoc derivative **1** was investigated. An incompatibility with the Triton X‐100 solution containing 1 % sodium dodecyl sulfate (SDS, **4**) was found. The staining pattern changed remarkably to larger, round signals with FWHM>5 pixels, which were not identified as K_Ca_3.1 channels. SDS (**4**) is a lipophilic and ionic agent, which is able to form micelles. We assume that the lipophilic BODIPY‐dye part of the small molecule **1** can be embedded into these micelles, which are then observed as bright, round signals. (Figure 8)

Changing the permeabilization method by using the non‐ionic surfactant saponine and subsequent incubation with BODIPY‐labeled senicapoc derivative **1** led to the expected punctate staining pattern of the K_Ca_3.1 channel with FWHM≤5 pixels. (Figure 3)

Co‐staining was performed in order to show colocalization of the signals obtained by indirect immunofluorescence and the BODIPY‐labeled senicapoc derivatives **1** and **2**. Starting with the indirect immunofluorescence protocol and subsequent incubation with the senicapoc derivatives **1** or **2** for 10 min (protocol 4A) led to signals using the TRITC filter set (showing antibody‐based staining), but no signals were observed in the FITC filter set displaying **1** or **2**. However, signals were observed using both filter sets after extending the incubation time with **1** or **2** to 20 min (protocol 4B). For this reason, the staining order was changed: After permeabilization, A549‐3R cells were incubated with the senicapoc derivatives **1** or **2** first and then with the antibodies for the antibody‐based indirect immunofluorescence (protocol 5). Signals were observed for both filter sets. The signals received from protocols 4B and 5 were analyzed with a line scan showing the intensity profile of the signals in x‐ and y‐direction using the MetaVue software. Signals were counted as colocalized when the difference of the maxima of two signals from the TRITC and the FITC filter set was ≤1 pixel^[14]^ (see supporting information). The results obtained from this analysis were surprising and unexpected. It was found that approx. 50 % of the signals were caused by the staining method, which was performed first. Approx. 45 % of the signals could be assigned to the second staining method and approx. 5 % of the signals were colocalized. If both methods labeled different targets, the density would have been higher than after staining with BODIPY‐labeled senicapoc derivatives **1** or **2** or antibody‐based indirect immunofluorescence only. This was not the case: Summing up all signals/μm^2^ (only red+only green+yellow dots) gave similar total numbers as obtained by staining with BODIPY‐labeled senicapoc derivatives **1** or **2** (protocol 1) or antibody‐based indirect immunofluorescence only as we had shown previously.^[12]^ These results led to the conclusion that only either the antibodies or the BODIPY‐labeled senicapoc derivatives **1** or **2** can bind to one K_Ca_3.1 channel protein.

**Figure 9:**
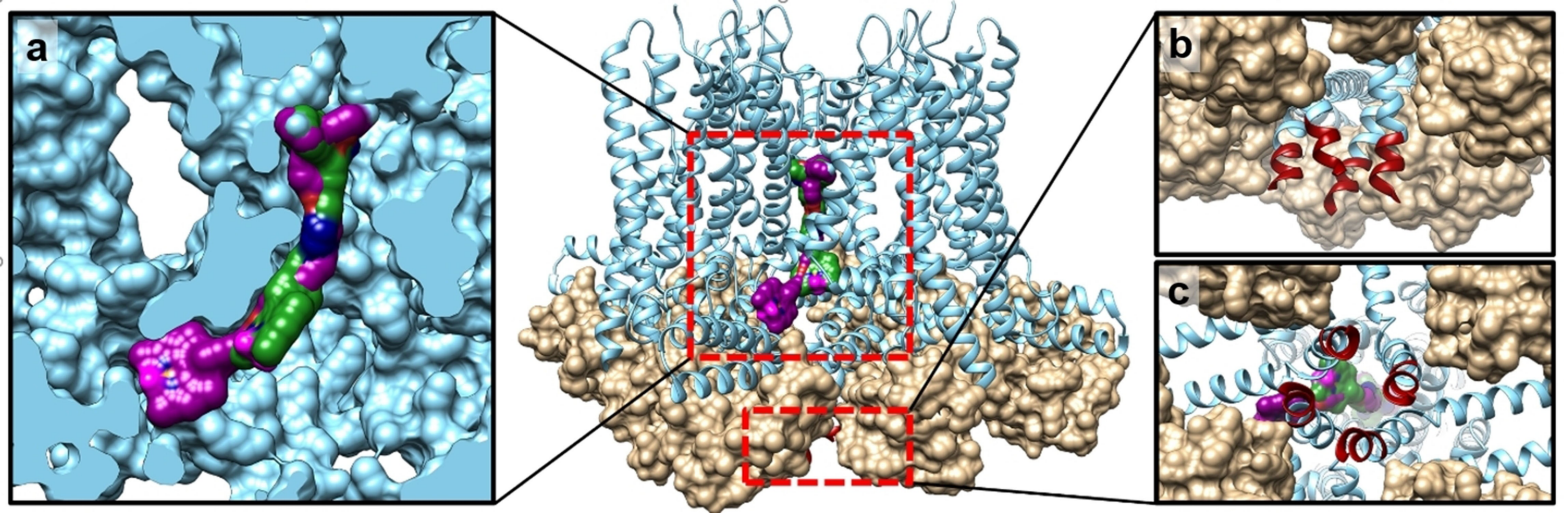
Binding of the BODIPY‐labeled senicapoc derivatives **1** (green) and **2** (magenta) and the antibody binding region (red) in K_Ca_3.1 (pdb 6cno ^[17]^, light blue: K_Ca_3.1 channel as ribbons, tan: calmodulin as surface representation). Section **a**) Docking‐based binding of **1** and **2** in the inner pore. The channel is shown as a clipped surface representation. Section **b‐**‐**c**) Different views on the starting sequence of the antibody binding region. The rest of the binding sequence is not solved in the EM structure. The figures were created using UCSF Chimera.^[18]^

These findings can be explained by the expected binding sites of the senicapoc derivatives and the antibody (Figure 9). It is accepted, that senicapoc and presumably also the BODIPY‐labeled senicapoc derivatives **1** and **2** bind to the inner pore in the open state of the ion channel as in the calmodulin‐bound open state (Figure 9a).^[12]^ Both compounds show a similar binding mode with the exception that the derivative **2** with the longer linker binds deeper into the binding pocket. Figure 9b/c shows a more detailed view of the binding region of the antibody. Unfortunately, only parts of this binding region are resolved in the K_Ca_3.1 structure. It can be seen that this region is partly shielded by the bound calmodulin, which could prevent antibody binding. Together with the experimental data, it can be postulated that different conformations are required for binding of the senicapoc derivatives and the antibody, and a co‐staining of both is therefore not possible.

It can be expected that the antibody binds to the closed conformation, since the inactive state is the more often occurring state. The maximal open probability (P_O,max_) of the K_Ca_3.1 channel at saturating Ca^2+^ concentration is 0.44.^[16]^ In addition, the binding region of the antibody seems to be blocked by the bound calmodulin. Thus, antibody binding at the ion channel should occur preferably in the closed conformation. In this conformation, the senicapoc derivatives **1** and **2** cannot bind to the channel.

These different conformations also allow interpreting the results of the co‐staining experiments. The cells were washed and fixed before staining, which may lead to an activation of the ion channels during the initial fixation process (P_O,max_=0.44).^[16]^ The senicapoc derivatives are binding to the open conformation, the antibody is binding to the closed conformation, and rarely binding of both can be identified. It therefore seems that the senicapoc derivatives and the antibody cannot bind simultaneously due to the different conformations needed for binding. The remaining approx. 5 % of colocalized signals can be explained by a remaining low affinity of the antibody for binding to the open conformation.

In order to confirm binding of the BODIPY‐labeled senicapoc derivatives **1** and **2** to the K_Ca_3.1 channel, patch‐clamp experiments were performed. An inhibition of the K_Ca_3.1 channel by compounds **1** and **2** was revealed by the significantly decreased current density after application of **1** or **2**. However, the BODIPY‐labeled senicapoc derivatives **1** and **2** did not reach the potency of senicapoc. Nonetheless, the significant decrease of the current density confirmed inhibition of the K_Ca_3.1 channel by the BODIPY‐labeled senicapoc derivatives **1** and **2** and, therefore, binding to the desired target.

## Conclusions

In summary, both BODIPY‐labeled senicapoc derivatives **1** and **2** represent novel small‐molecule fluorescent probes for fast, efficient and selective imaging of K_Ca_3.1 channels. Docking experiments explain that co‐staining with the antibody‐based indirect immunofluorescence is not possible due to different conformations needed for binding of the senicapoc derivatives and the antibody. Thus, the antibody used here and compounds **1** and **2** label different channel populations, namely, closed and open channels, respectively. Patch‐clamp experiments show inhibition of the K_Ca_3.1 channel by compounds **1** and **2** confirming binding of **1** and **2** to the K_Ca_3.1 channel. However, labeling with **1** having only one OCH_2_CH_2_ moiety in the linker between the targeting component and the BODIPY dye resulted in images with better signal‐to‐noise ratio. Thus, the punctate staining pattern of the K_Ca_3.1 channel appeared more crisp and images were easier to analyze.

## Experimental Section

*Materials*. 10 mM stock solutions of **1** and **2** were prepared in a glass vial with DMSO as solvent. The staining solutions of **1** and **2** (10 μM) were obtained by diluting the stock solutions with phosphate buffered saline (PBS, 1 : 1000) in a microcentrifuge tube (Eppendorf®) and dissolving the precipitate by using a Vortex (Scientific®) for 5 min. For the indirect immunofluorescence assay we used normal goat serum (Sigma Aldrich, 10 %, Na_2_HPO_4_ (0,01 M) und NaCl (0,15 M) in Ampuwa^TM^), anti‐K_Ca_3.1 antibody produced in rabbit (Sigma Aldrich, AV35098, binding sequence: “DLQQNLSSSHRALEKQIDTLAGKLDALTELLSTALGPRQLPEPSQQSK”) and Cy3‐conjugated goat anti‐rabbit IgG secondary antibodies.

*Cell culture*. We used the A549‐3R cell line, a hypotriploid epithelial cell line from a non‐small‐cell lung cancer (NSCLC) taken from a 58‐year‐old Caucasian in 1972. The suffix “3R” refers to the repeated process of intravenous administration of parental cells (also initially “0R”) into immunocompromised mice to obtain lung metastases. Cells from these metastases were isolated and cultured *in vitro* followed by another round of reinjection to select for tumor cells with high metastatic potential.^[19]^ A549‐3R cells overexpress K_Ca_3.1 channels and thus are suitable for corresponding staining experiments.^[7]^ The cells were cultured in Dulbecco's Modified Eagle's Medium (DMEM) with 4.5 g/L glucose and supplemented with 10 % fetal calf serum (FCS Superior) at 37 °C and 5 % carbon dioxide (CO_2_) in cell culture dishes (Ø=10 cm). For experiments, cover slips were coated with 0.1 % poly‐L‐lysine (30 min, room temperature) and washed with Dulbecco's Phosphate Buffered Saline (PBS). One cover slip was placed in each well of a 12‐well‐plate. DMEM (1 mL) was added to each well and approximately 100.000 cells were added. The cells were incubated overnight at 37 °C and 5 % CO_2_ prior to the experiments.

*Staining protocols*. For each experiment, the medium was removed and the cells were washed three times with PBS and fixed with 3.5 % paraformaldehyde (PFA) in PBS for 30 min at room temperature. Then the cells were washed three times with PBS at room temperature and kept for 10 min in PBS supplemented with 100 mmol/L glycine and washed again three times. Thereafter, the staining protocols were performed:

Protocol 1: A drop (30 μL) of the staining solution of **1** or **2** (10 μM in PBS) was pipetted onto Parafilm. The cover slip with the adherent A549‐3R cells was placed carefully upside down onto the drop and the NSCLC cells were incubated in a humidified dark chamber for 10 min. After washing five times with PBS, the cover slip was placed upside down onto a microscope slide.

Protocol 2: The cells were incubated with Triton X‐100 (0.25 % Octoxinol 9 in 1 % SDS/PBS) in PBS for 10 min at room temperature. After washing five times with PBS, staining protocol 1 was performed as described above.

Protocol 3: The cells were incubated with saponine solution (0.5 % in PBS) for 20 min at room temperature. After washing five times with PBS, staining protocol 1 was performed as mentioned above.

Protocol 4: The cells were incubated with saponine solution (0.5 % in PBS) for 20 min at room temperature. After washing five times with PBS, the cover slip was carefully placed upside down onto a 30 μL drop of normal goat serum on Paraflim and incubated in a moist, dark chamber for 30 min to block unspecific binding of the antibodies. Thereafter, the cover slips were placed upside down on a 30 μL drop of primary antibodies in normal goat serum (1 : 300; anti‐K_Ca_3.1 antibody) on Parafilm and incubated for 2 h. The cells were then washed five times with PBS and incubated with Cy3‐conjugated secondary antibodies in normal goat serum (1 : 500; goat anti‐rabbit IgG, 30 μL drop on Parafilm) for 1 h at room temperature. At the same time, negative controls were incubated with only Cy3‐conjugated secondary antibodies (1 : 500; goat anti‐rabbit IgG). After washing five times with PBS, the cells were fixed with 3.5 % PFA in PBS for 10 min at room temperature and washed three times with PBS. Thereafter, staining protocol 1 was performed with the same coverslips as detailed above (incubation time: protocol 4 A: 10 min; protocol 4B: 20 min).

Protocol 5: The cells were incubated with saponine solution (0.5 % in PBS) for 20 min at room temperature. After washing five times with PBS, staining protocol 1 was performed as detailed above. Thereafter, the antibody‐based indirect immunofluorescence staining was performed as described in the preceding protocol 4. After the final fixation with 3.5 % PFA in PBS for 10 min at room temperature and washing three times with PBS, the cover slip was placed upside down onto an object slide for microscopy.

*Microscopy*. Microscopy was performed with an inverted microscope (Axiovert 200, Zeiss AG, Oberkochen, Germany) equipped with a x100 oil immersion objective and a digital camera (SPOT RT SE from Diagnostic Instruments Inc.). Filters were used to irradiate fluorescent dyes with light according to their absorption maxima and to filter adequate wavelengths of emitted light. The following filter sets were used: FITC (Carl Zeiss filter set 10, bandwidth excitation filter 450–490, dichroic mirror 510, bandpass emission filter 515–565) and TRITC (Carl Zeiss Filter Set 15, Bandpass Filter 546/12, dichroic mirror 580, emission filter Long Pass 590). Signals detected in the FITC and TRITC channels are denoted as “green” and “red” fluorescence, respectively. The senicapoc‐based staining with imaging probes **1** and **2** was recorded in the FITC (green) channel and the antibody‐based staining in the TRITC (red) channel. Data acquisition and analysis were performed with Metavue software (version 6.3r6 from Molecular Devices LLC, Visitron). Signals with a *Full Width at Half Maximum* ≤5 pixels (∼300 nm) were regarded as a single K_Ca_3.1 channel.

For the evaluation of colocalization of the signals of BODIPY‐labeled senicapoc derivatives **1** or **2** and the indirect immunofluorescence, ten squares with a side length of 50 pixels, corresponding to an area of 9 μm^2^, were randomly scanned in a given cell. When a signal was seen in both fluorescence channels, colocalization of the signals required that the x and y coordinates of the “red” and “green” maxima did not differ by more than one pixel.^[14]^ Colocalized signals are denoted as “yellow”. The experiments were repeated with three independent passages of A549‐3R cells (N=3), and n=5 cells were analyzed at a time.

*Patch‐clamp experiments*. A549‐3R cells were used for the experiments. Whole cell recordings were performed at room temperature using borosilicate glass pipettes (GC150TF‐10, Harvard Ltd., USA) connected to an EPC‐10 amplifier (HEKA Electronics, Lambrecht, Germany). The typical electrode resistance was 3–4 MΩ, while series resistance was in the range of 5–15 MΩ. Series resistance compensation of >30 % was routinely used. Voltage‐clamp experiments on cultured cells were controlled by PatchMaster software (HEKA Electronics, Germany). Current density was calculated by dividing the current amplitude determined at the end of the depolarizing voltage ramp to +60 mV by the membrane capacitance obtained from slow capacitance compensation. The following recording solutions were used: 1) Extracellular solution (mM): 140 NaCl, 5 KCl, 10 4‐(2‐hydroxyethyl)‐1‐piperazineethanesulfonic acid (HEPES), 1 MgCl_2_, and 1 CaCl_2_, pH=7.4 with NaOH. 2) Intracellular solution (mM): 140 KCl, 10 HEPES, 1.3 ethylene glycol‐bis(β‐aminoethyl ether)‐N,N,N′,N′‐tetraacetic acid (EGTA), 1.217 CaCl_2_, and 1 MgCl_2_, pH=7.4 with KOH. The calculated free Ca^2+^ concentration of the internal solution was 1 μM in order to obtain full activation of the K_Ca_3.1 channels during the patch clamp experiments. 1‐EBIO (50 μM), **1** (10 μM), **2** (10 μM) and senicapoc (1 μM) were dissolved in DMSO and added to the standard extracellular solution and applied in this order for a period of 2–5 min. The final DMSO concentration was ≤0.15 %. A multibarrel application pipette with a tip diameter of about 100 μm was used for test substance application close to the recorded cell. Recordings were analyzed using FitMaster and Excel software.

*Statistical analysis*. Data are given as mean±SEM. ANOVA was performed followed by t‐testing for paired experiments. The analysis was done using Excel software. Statistical difference was determined with the t‐test (p<0.05).

## Conflict of interest

The authors declare no conflict of interest.

## Supporting information

As a service to our authors and readers, this journal provides supporting information supplied by the authors. Such materials are peer reviewed and may be re‐organized for online delivery, but are not copy‐edited or typeset. Technical support issues arising from supporting information (other than missing files) should be addressed to the authors.

Supplementary
