# Supplementary material for: Co‐staining of KCa3.1 Channels in NSCLC Cells with a Small‐Molecule Fluorescent Probe and Antibody‐Based Indirect Immunofluorescence
Source: ChemMedChem. 2020 Oct 21;15(24):2462–9. doi: 10.1002/cmdc.202000652 (PMC7756743; doi:10.1002/cmdc.202000652)
Supplement: Supplementary file 1 — Supplementary [file CMDC-15-2462-s001.pdf]

ChemMedChem

Supporting Information

# Co-staining of $K_{Ca}3.1$ Channels in NSCLC Cells with a Small-Molecule Fluorescent Probe and Antibody-Based Indirect Immunofluorescence

Kathrin Brömmel, Sarah Maskri, Etmar Bulk, Zoltan Pethő, Marius Rieke, Thomas Budde, Oliver Koch, Albrecht Schwab, and Bernhard Wünsch*

# Supporting Information

## Contents

Analysis of the $K_{Ca}3.1$ channel density……………………………………………….....S2

Analysis of colocalization……………………………………………………………….S5

Results of colocalization ….…….……………………………...……………………S6

Docking……………………………………………………………...……………………S7

References………………………………………………………………………...…..S8

**Analysis of the $K_{Ca}$3.1 channel density**

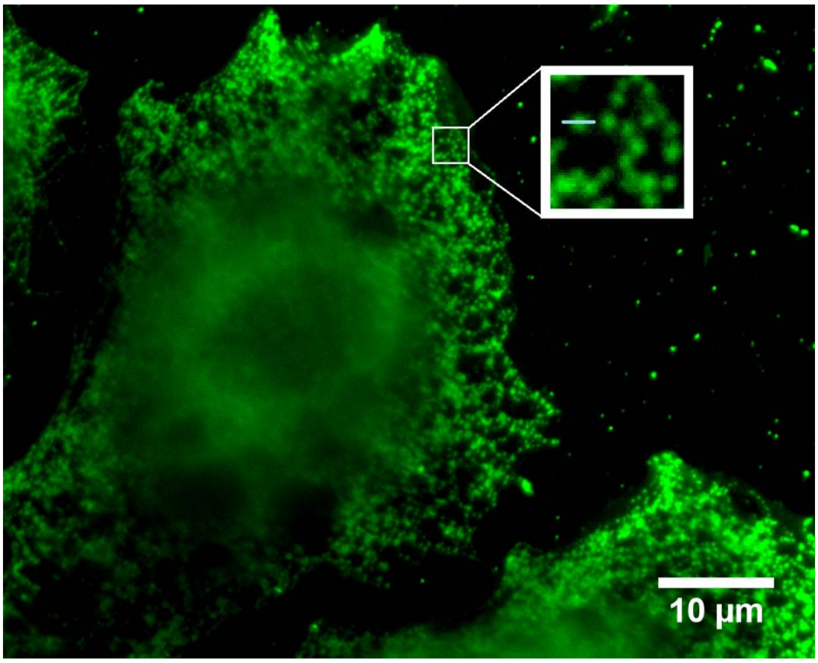


Figure S1: Signal analysis with linescans: NSCLC cells incubated with a 10 µm staining solution of BODIPY-labeled senicapoc-derivative **1** (white box: 50 x 50 pixel) with linescan (blue line).

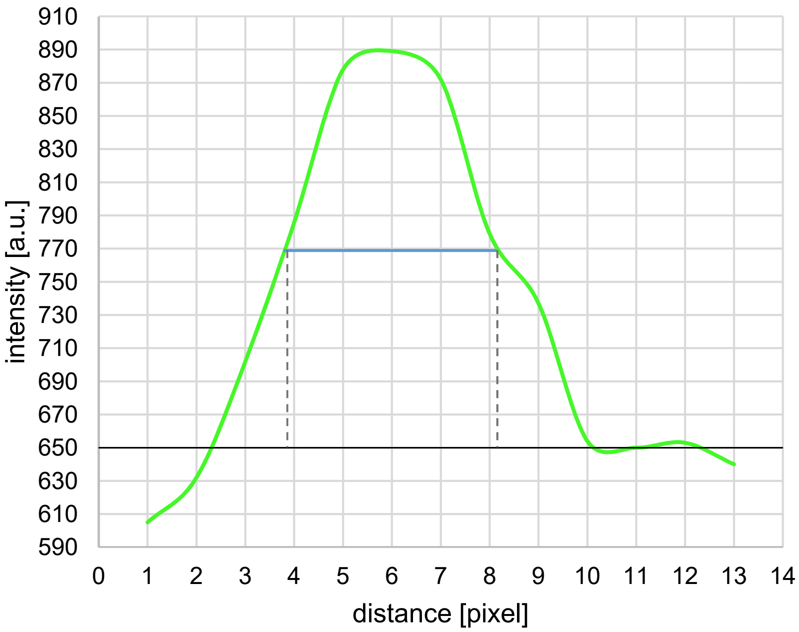


Figure S2: Intensity profil of the linescan in Figure S1. Y axis: intensity in arbitrary units. X-axis: distance in pixel.

At first the zero line is set (black; ($y_{min}$ = 650)). The *Full Width at Half Maximum* (FWHM, $y_{1/2}$) was calculated for each peak by the following formula:

$$y_{1/2} = [0{,}5(I_{max} + I_{min})]$$

In this example:

Peak: $I_{min}$ = 650 a.u. ; $I_{max}$ = 889 a.u.: $y_{1/2}$ = [0,5 (650 + 889) = 769,5 a.u.

Next, the difference of the x-values for $y_{1/2}$ were calculated, which is the FWHM.

(Table S1)

Table S1: Calculation of FWHM

| $y_{1/2}$ [a.u.] | $x_1$ [pixel] | $x_2$ [pixel] | FWHM [pixel] |
|---|---|---|---|
| 769,5 | 3,8 | 8,2 | 4,7 |

Signals with FWHM ≤ 5 pixel were counted as $K_{Ca}$31. channel.

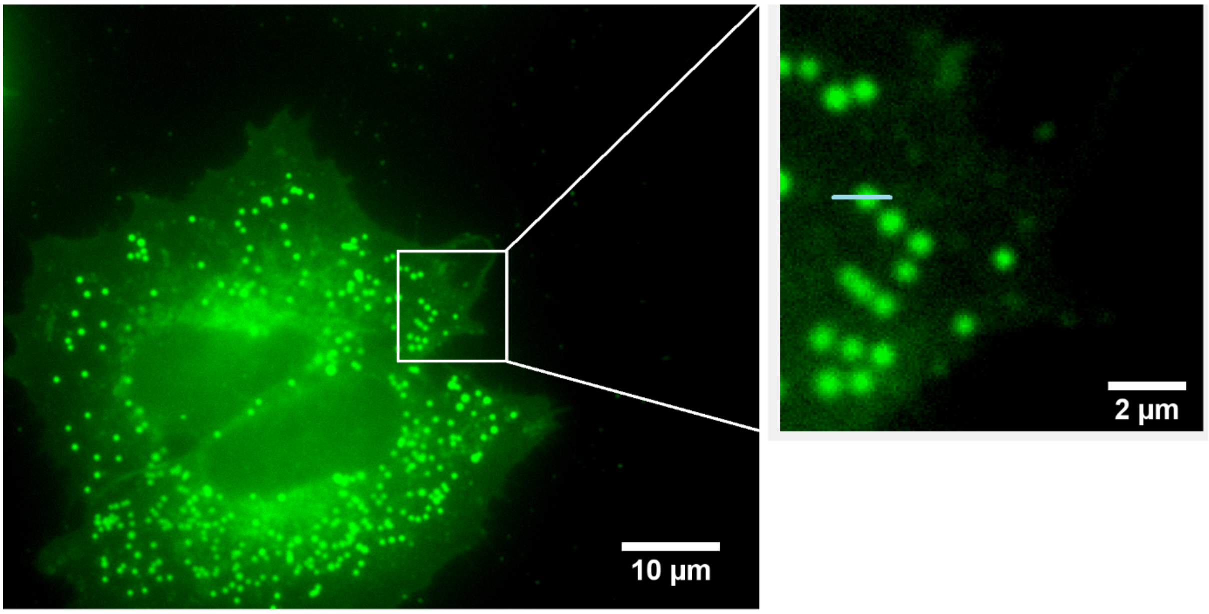


Figure S3: Signal analysis with linescans: NSCLC cells permeabillized with Triton$^{TM}$ X-100 solution and incubated with a 10 µm staining solution of BODIPY-labeled senicapoc-derivative **1** with linescan (blue line).

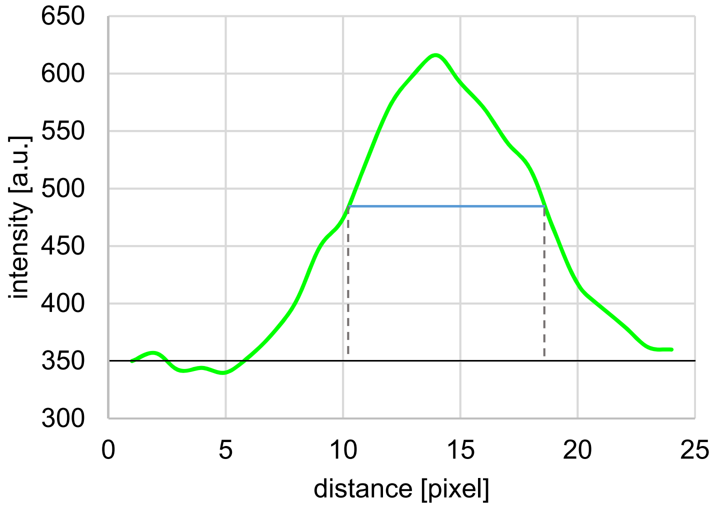


Figure S4: Intensity profil of the linescan in Figure S3. Y axis: intensity in arbitrary units. X-axis: distance in pixel.

FWHM >> 5 pixel.

## Analysis of colocalization

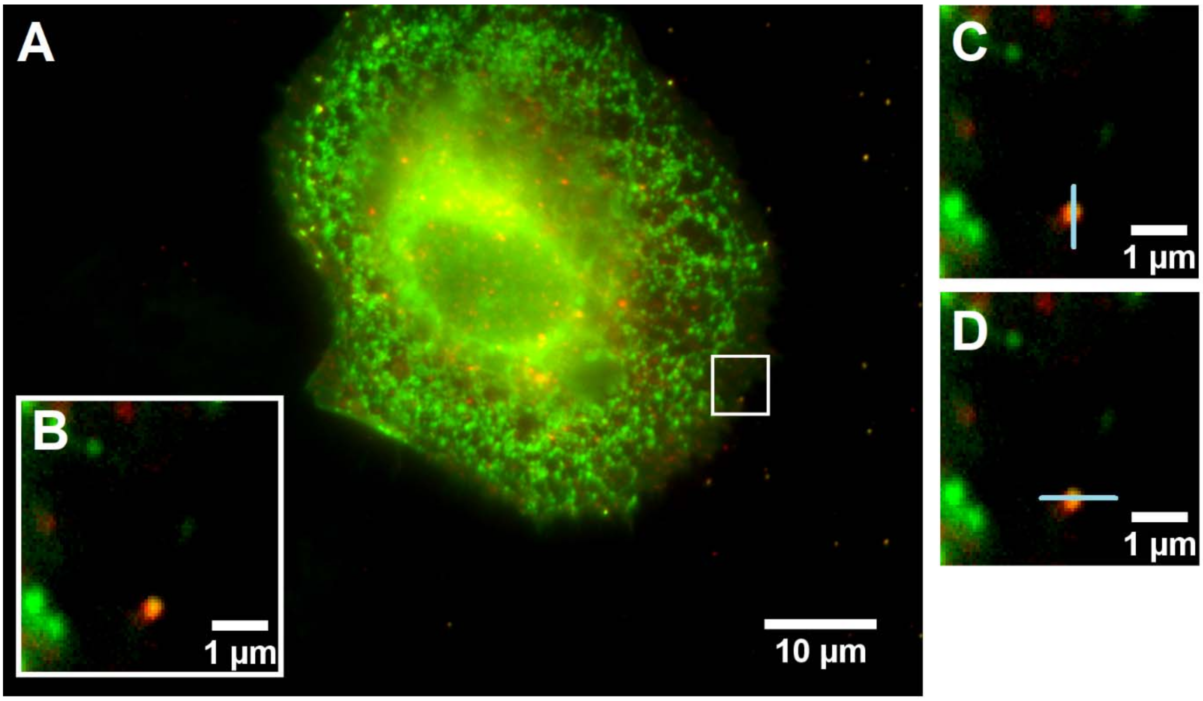


Figure S5: Signal analysis with linescans: NSCLC cells after co-staining (protocol 4B, BODIPY-labeled senocapoc-derivative **1**) with vertical and horizontal linescan (blue line).

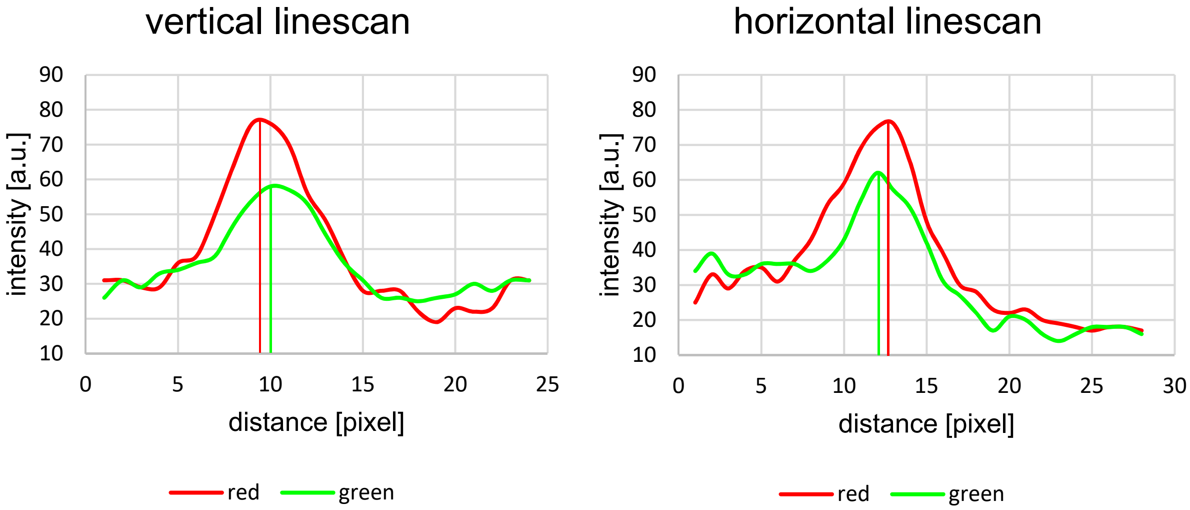


Figure S6: Intensity profils of the linescans in Figure S5. Y axis: intensity in arbitrary units. X-axis: distance in pixel.

Colocalization: The Maxima of the "red" and "green" linescan do not differ by more than one pixel.

## Analysis of colocalization

Table S2: Relative frequency of channel labelling.

| **protocol - compound** | **yellow [%] ± SEM** | **only red [%] ± SEM** | **only green [%] ± SEM** |
|---|---|---|---|
| 4B - **1** | 6.0 ± 0.4 | 51.6 ± 0.3 | 42.4 ± 0.3 |
| 5 - **1** | 5.3 ± 0.3 | 45.2 ± 1.0 | 49.5 ± 0.9 |
| 4B - **2** | 4.7 ± 0.3 | 51.0 ± 0.3 | 44.3 ± 0.4 |
| 5 - **2** | 4.3 ± 0.4 | 45.2 ± 0.2 | 50.5 ± 0.4 |

**Docking**

The modelling was based on the available Cryo-EM structure (pdb 6cno [1]) and performed with the Molecular Operating Environment program version 2019 (MOE) [2]. At the beginning, a structure preparation was performed including the 3D protonation of the protein (at pH 7.4), deleting distant waters, tethering and fixing atoms, and refining the structure by adjusting and adding missing residues and loop segments. Gold version 5.7.1 [3] was used for docking. The binding site was defined based on a point in the inner pore and a radius of 24Å for derivative 1 and 30 Å for 2. The ligands were docked using Goldscore and rescored using ChemPLP. The early termination was switched off and the 100 diverse solutions were created. An automatic search efficiency of 200% was used. The options " Flip pyramidal N, flip amide bond and flip ring corners" were also switched on. Unfortunately, GOLD is unable to deduce the atom type of the bore atom while docking. Therefore, the bore and some heterocycle atoms were modified during docking while retaining aromaticity and planarity. After docking, the selected poses were modified into the original dye following a minimization in MOE afterwards.

## References

[1] C.-H. Lee, R. MacKinnon, *Science*, **2018**, *360*, 508-513.

[2] Molecular Operating Environment (MOE), 2019.01; Chemical Computing Group ULC, 1010 Sherbrooke St. West, Suite #910, Montreal, QC, Canada, H3A 2R7, 2019.

[3] G. Jones, P. Willett, R. C. Glen, A. R. Leach, R. Taylor, *J. Mol. Biol.*, **1997**, *267*, 727-748.
